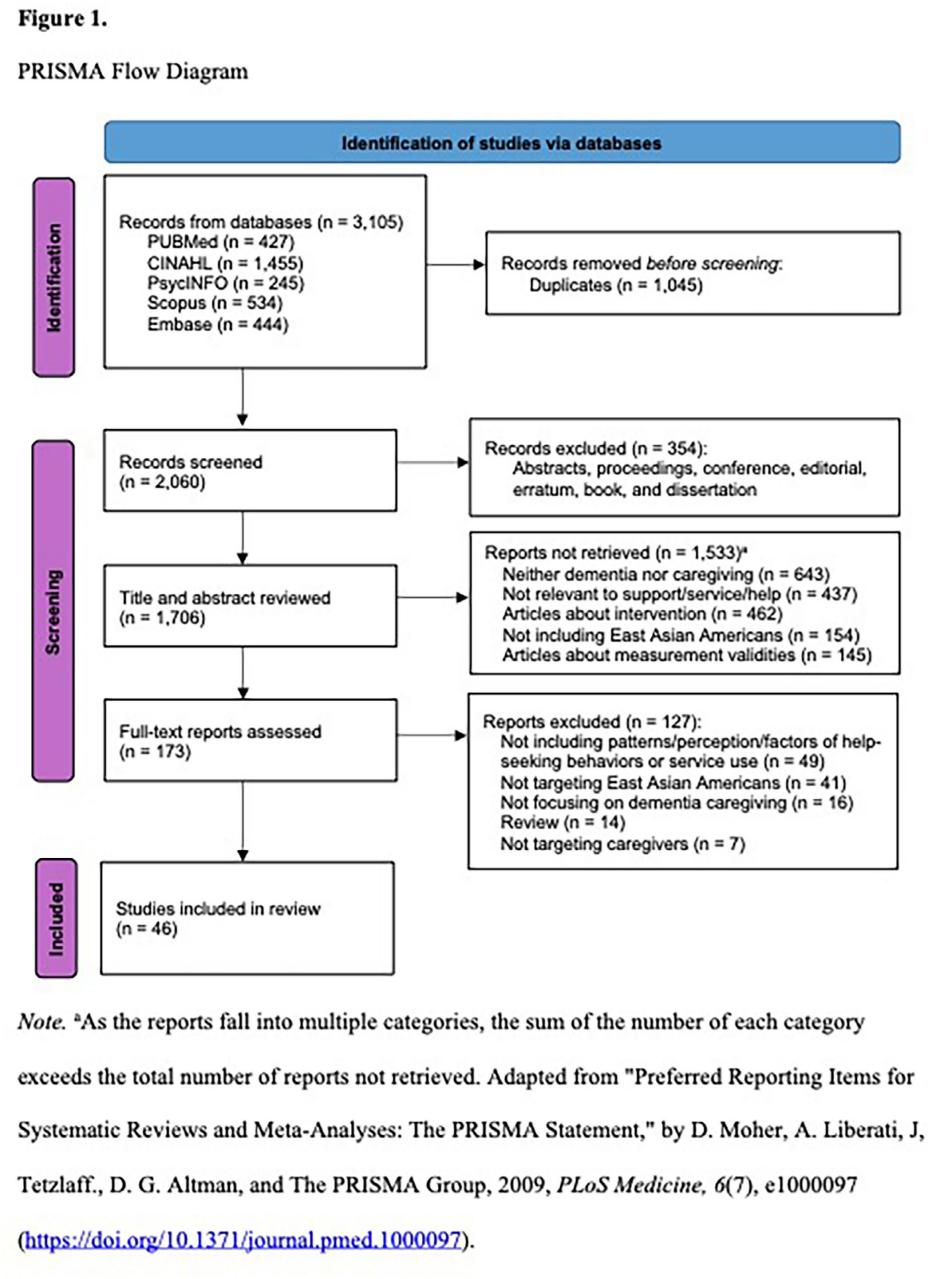# Help‐Seeking Behaviors in Dementia Caregivers of East Asian Backgrounds: An Integrative Review

**DOI:** 10.1002/alz70858_097776

**Published:** 2025-12-24

**Authors:** Eunjung Ko, Sooyoung Kim, Bei Wu

**Affiliations:** ^1^ New York University Rory Meyers College of Nursing, New York, NY, USA; ^2^ The Ohio State University College of Medicine, Columbus, OH, USA; ^3^ New York University, New York, NY, USA

## Abstract

**Background:**

Asians are heterogeneous, underscoring the need to examine their subgroups. With the global rise in the older Asian population, the number of Asians living with dementia is expected to exceed 80 million by 2050, including approximately 36 million East Asians. While dementia caregivers of East Asian backgrounds often face high levels of caregiving obligation and stress, their cultural values, such as filial piety and stigma, may hinder seeking support. Still, research on help‐seeking behaviors within Asian subgroups remains limited. This review aimed to identify help‐seeking behaviors among dementia caregivers of East Asian backgrounds.

**Method:**

Articles were searched between July and September 2024 through six databases: PubMed, CINAHL, PsycINFO, Scopus, Embase, and Web of Science. Articles were included if they were published in English and targeted dementia caregivers of East Asian backgrounds and help‐seeking behaviors. Thematic analysis was used to synthesize the themes. Quality assessment was conducted using JBI tools and MMAT.

**Results:**

We included 46 articles (31 qualitative, 13 quantitative, and 2 mixed methods) published between 1998 and 2024. Nineteen were conducted in East Asia, with the others in North America and Oceania. Four themes emerged: (1) patterns of help‐seeking behaviors (stages of help‐seeking, sources of support, cultural values, and priority for dementia care), (2) facilitators and barriers (individual, familial, organizational, and cultural factors), (3) coping mechanisms (emotion‐focused vs. problem‐focused and adaptive vs. maladaptive), and (4) unmet needs of formal support (increasing awareness, improving accessibility, and support for dementia caregivers). Dementia caregivers of East Asian backgrounds preferred in‐home care for their care recipients due to a filial obligation. They relied on informal networks—such as family, neighbors, and churches—for practical support. While they used formal services to obtain information about the disease and available care options, limited accessibility, insufficient culturally adapted resources, and stigma hindered seeking formal support. The assessed quality of each article varied.

**Conclusion:**

This review's findings show how previous articles addressed the help‐seeking behaviors among dementia caregivers of East Asian backgrounds. Given their unmet needs reported in the literature, it is pivotal to explore strategies to address these gaps, such as offering culturally tailored programs.